# Uncovering patients’ preferences for brand among essential classes of coronary heart disease medications using a discrete choice experiment

**DOI:** 10.1038/s41598-024-77007-3

**Published:** 2024-11-04

**Authors:** Ehab Elmongui, Ghada Abu-Sheasha, Adel Zaki, Omaima Gaber Yassine, Asmaa Abd Elhameed

**Affiliations:** 1 Medical Research Institute, Alexandria University, 165, Horreya Avenue, Hadara, Alexandria, Egypt; 2 Health Insurance Organization, Alexandria, Egypt

**Keywords:** Patient preference, Relative importance, Discrete choice experiment, Coronary heart disease, Brand, Generic, Health insurance, Therapeutics, Cardiology, Outcomes research

## Abstract

Patient preferences for medications strongly correlate with adherence; one area of interest is the choice between branded and generic drugs. Despite extensive research about brand-versus-generic drug preferences, few studies have explored severe-illness patients like those with coronary heart disease (CHD). We could not locate studies measuring preference weights of branded drugs in different classes within guideline-recommended regimens using discrete choice experiments (DCE). We aimed to explore the preference for branded medications used for secondary prevention of CHD events among patients receiving treatment at one of the largest Egyptian health insurance clinics. Patients with CHD were interviewed to choose between various therapy regimens containing brand-name and generic versions of aspirin, beta-blockers, statins, and renin–angiotensin–aldosterone system blockers (RAAS blockers). The study employed a DCE technique and followed the recommendations of the International Society of Pharmacoeconomics and Outcomes Research (ISPOR). Seventy-two percent (149) of the 208 patients questioned were dissatisfied with at least one of their generic medications. The majority of unsatisfied patients displayed brand preferences across the four medicine classes, primarily due to the notion that generics may be less effective. Patients preferred the RAAS blocker brand the most (adjusted odds ratio [AOR]: 3.14; 95% confidence interval [CI] 2.83 to 3.48), followed by beta-blockers (AOR: 2.06; 95% CI 1.88 to 2.27) and statins (AOR: 1.5; 95% CI 1.50 to 1.61). The relative importance of each class from the patient’s perspective showed the highest importance with RAAS blockers (22.2%) and beta-blockers (14.1%), while statins and aspirin had minor importance (7.8% and 6.6%, respectively). In the present study, branded drugs for secondary CHD prevention were preferred over generic alternatives. This finding has two implications for clinical practice. Firstly, physicians and pharmacists need to assure patients about the quality of generics to insure patient satisfaction and adherence to medication. Secondly, health insurance providers need to confirm the effectiveness of generics through observational studies. Despite the well-proven protective effects of aspirin and statins, they had minor importance from the patient’s perspective, highlighting the need to enhance patient knowledge. DCE was demonstrated to be a useful tool for eliciting the genuine preferences of patients treated within the setting of health insurance.

## Introduction

Patient preferences for their medications substantially impact how well they are taken and how patients perceive the quality of their medicines affect their adherence. Preferences for branded and generic pharmaceuticals are one area of significant study interest. A recent randomized controlled trial showed that even when patients express positive opinions about the quality of generic medications, generic labeling may have a negative impact on treatment adherence^1^. Generic medicines are bioequivalent to original, branded medicines. They should have the same strengths, dosage, efficacy, and safety as the original versions. Nevertheless, because they are sold for a significant discount, health insurance systems may be able to use them to increase the cost-effectiveness of their drug formularies.

Fifty-eight percent of the Egyptian population is covered by the Health Insurance Organization (HIO), an employment-based health insurance program. However, the nation switched to a new universal health care system beginning in 2018, whose law was issued in 2018 and shall be implemented gradually in 6 phases, with completion anticipated by 2032^2^. The HIO prescription requirements, like any health insurance system, require prescribing drugs with generic names to reduce healthcare costs. By Law 79, the public health insurance system changed from one that required premiums and copayments to one that just required premiums (from salary contributions) and no copayments for prescription medications^3^. The HIO offers an optional service that allows patients to pay the price difference to get the brand if offered in its owned pharmacies, even though the HIO relies on generic drugs^3^. This out-of-pocket money is offered at significantly lower prices than the brands’ prices in the private market because of the generic price deduction and because HIO purchases drugs with high discounts through the Egyptian Authority for United Procurement, Medical Supply, and The Management of Medical Technology^4^.

Most global studies comparing generic and branded medications have looked at public awareness, attitudes, and acceptability of generic alternatives. Few studies have looked at patient populations, such as those with serious illnesses. Most of those studies investigated a single class of medications like anti-epileptics^5,6^ antiretrovirals^7,8^, cyclosporin^9^, and atypical antipsychotics^10^. One study using mail survey in USA found that using generic drugs for heart conditions were associated with the highest risk rating by the consumers compared to other conditions (high blood pressure, strep throat, pain, and cough)^11^. None of those studies compared the relative importance of choosing branded drugs versus generic alternatives within different pharmacological classes, in terms of their impact on the utility of a standard therapeutic regimen. We could not locate studies that applied the discrete choice experiment (DCE) approach to measure the preference weights of the branded medications from various classes that were part of a suggested therapy regimen in guidelines.

One of the leading causes of death worldwide is coronary heart disease (CHD). There is limited information about patient preferences for branded and generic CHD medications in Egyptian health insurance clinics. Decision-makers would be better served if they were to comprehend the patients’ perspectives to encourage drug adherence and accomplish desired therapeutic goals.

Our aim in this study is to use a DCE to evaluate the stated patient preferences for the branded and generic forms of the four classes of medications that are frequently used for the secondary prevention of CHD: statins, beta-blockers, renin–angiotensin–aldosterone-system blockers (RAAS blockers), and aspirin, in a cohort of patients with national insurance.

## Methods

We conducted a DCE in accordance with the relevant guidelines and regulations provided by the International Society of Pharmacoeconomics and Outcomes Research (ISPOR) good research practices that aimed to standardize the methods used for conjoint analysis in healthcare research^12^. Each respondent was asked to answer ten tasks; each task consisted of a multiple-choice question with three alternatives from which he had to select the one that perceived maximizing the utility from his perspective. Each alternative had a different profile based on the levels of the attributes. The alternative’s attributes consisted of a set of drug classes (a whole regimen), and their levels were whether the drug class was a brand or generic.

### Study population

The study included a sample of adult nationally insured CHD patients in Alexandria, Egypt, who regularly come to the health insurance clinics in Alexandria to get their medicines. Alexandria has one of the highest HIO coverage in Egypt at 77.2% which indicates the importance of studies at HIO institutions as they represent an important sector of Alexandrian population^13^. The study included the following drugs given for the secondary prevention of CHD: hydroxyglutaryl-CoA reductase inhibitors (statins), beta-blockers, RAAS blockers, and aspirin. The HIO offers all generics free of charge and the brands for a charge calculated from the difference between the procurement costs of the brands and their generics. Patients with psychological problems or mental disabilities were excluded.

The following methodological sections address the recommended ISPOR checklist for conjoint analysis in health care including the attributes and levels, construction of tasks, experimental design, preference elicitation, instrument design, data collection, and statistical analyses. In addition, a section describing how drivers for brand inclination was assessed is included before the statistical analyses.

### Identification and selection of attributes and their levels

We identified six classes with proven benefits in reducing cardiovascular morbidity and mortality; the list included statins, beta-blockers, RAAS blockers, antiplatelets (PGY12 inhibitors and aspirin), and aldosterone antagonists^14–16^. After reviewing the prescription norms in HIO, we found that aspirin-PGY12 inhibitor dual antiplatelet therapy was prescribed for only one year post-acute coronary syndrome (post-ACS), and patients were de-escalated to aspirin alone. Additionally, we found that a minority were prescribed an aldosterone antagonist as indicated in CHD only for patients with LVEF ≤ 40% post-ACS who received a beta-blocker and ACE inhibitor. Therefore, we only included the four agents prescribed to most CHD patients: statins, beta-blockers, RAAS blockers, and aspirin. Each selected attribute (drug class) was set to have one of two levels (brand or generic).

A price for each alternative was calculated based on the classes whose levels were set to brands. Each class’s brand had a different price representing the average out-of-pocket amount: the procurement prices of the class’s brands minus the class’s generic procurement price available in the HIO. Since we had four classes, each with two levels, this resulted in 16 (2^4) different alternatives (profiles) with 16 different prices ranging from EGP 0 (when all classes’ levels were set to “generic”) to EGP 285 (when all classes’ levels were set to “brand”). The sixteen possible prices set for each profile according to the combination of class levels were EGP 0, EGP 22, EGP 41, EGP 63, EGP 68, EGP 90, EGP 109, EGP 131, EGP 154, EGP 176, EGP 195, EGP 217, EGP 222, EGP 263, EGP 244, and EGP 285.

### Construction of tasks

Each task consisted of three alternatives: two generated by the Conjointly software, and the third one was the status quo of the patient. The status quo is a term used to describe the respondent’s current situation; in our study, the status quo represents the patient’s combination set of the free-of-charge generics and the out-of-pocket brands he purchases.

### Experimental design

We used a D-efficient main effects design generated by Conjoint.ly software. This fractional factorial design has optimized orthogonality, level balance, and minimal overlap. The design included 16 blocks randomly assigned to the respondents, and each block consisted of 10 choice tasks without the price attribute. The total number of alternatives in the 16 blocks was 320 (20 alternatives per block). We then calculated the price of each of the 320 alternatives according to the classes of medicines whose levels were set to “brand”; this represented a realistic price and aimed to prevent the generation of choices with unbalanced utilities. Finally, we added the status quo for each respondent to each choice task during the interview.

### Preference elicitation

**Fig. 1:**
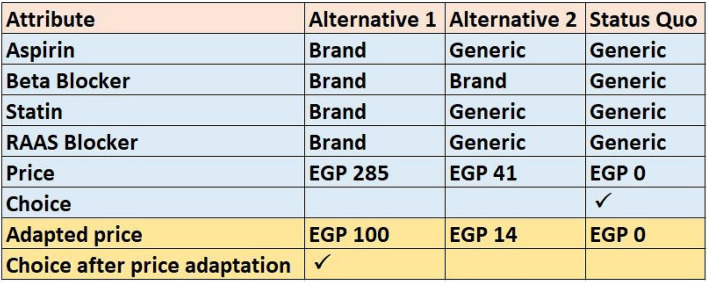
Example of an answered choice task before and after price adaptation.

To increase the statistical efficiency of our design, we presented two versions for each choice task: an initial version in which the prices were set as mentioned in the construction of tasks section and an adapted version where the prices of the two software-generated alternatives were decreased or increased depending on the alternative chosen in the initial version. If the respondent chose the cheapest alternative in the initial version, we asked the respondent to decrease the prices of the two unchosen alternatives down to a price that would change his initial choice to one of the originally unchosen alternatives. In contrast, if the respondent chose the most expensive alternative, we asked him to state the maximum price of this alternative he would pay. Figure 1 illustrates an example of an initial choice task before price adaptation from which the respondent chose the alternative that maximized the utility from his perspective; here, the respondent chose the cheapest alternative (with all classes’ levels set to “generic”, marked with ↑) and the adapted version where the prices of the two originally unchosen alternatives were reduced until he changed his choice to one of them.

### Instrument design and data collection

The choice set cards were constructed using visual aids. The interviewer manually distributed the actual packs of the medicines into two groups representing the two alternatives on each card. Each group included only the classes whose levels were set to “brand” in the alternative. The interviewer asked the patient whether he preferred the brands of group 1 priced at price x or to get the brands of group 2 at price y more than his status quo. The interviewer recorded the responses to each task on the data collection sheet corresponding to the block without exhausting the patient. We repeated one of the tasks to check the consistency and quality of the responses; only results with consistent choices were included in the analysis.

### Drivers for brand inclinations

We surveyed the patients for their drivers of brand inclination for each of the four CHD medications; we first identified the respondents who were not satisfied with the generic and then asked them to state the cause of dissatisfaction that drove them to be inclined to the brand. The drivers of brand inclinations were grouped into two general drivers: internal and external. The internal drivers included the insufficient efficacy of the generic, the side effects of the generic, or the general belief in the better quality of the brands. The external drivers included the advice of an HIO physician, other physicians, or a friend or relative.

### Sample size calculation

The sample size calculation was done using Conjointly software^17^ by simply entering the attributes (five attributes), levels (brand or generic), number of questions per participant (ten choice sets), and the number of alternatives in each question (two alternatives per choice set). This resulted in a minimum required sample size of 100 participants for the main analysis. We targeted 208 respondents (13 respondents for each block of the 16 blocks) to allow for subgroup analyses.

### Statistical analysis

Categorical variables were described using frequencies and percentages. Continuous variables were described using the mean and standard deviation. Independent t tests and chi-squared tests were performed to compare the sociodemographic characteristics of the satisfied and dissatisfied respondents.

Conditional logistic regression was used to estimate patients’ preference weights for the classes’ brands, the attribute part-worth utility of the whole class, and the willingness to pay (WTP) for the brand of each class of medicines. CHD classes’ levels had only two levels: brand and generic, where the generic was the reference. The preference weights *β* of the classes represent the change in the utility from generic to brand. For the price attribute, *β* represents the change in utility for every one-unit increase in the price of the regimen. Positive preference weights mean the brand is more preferred than the generic, and negative weights mean the opposite. The adjusted odds ratio (AOR) was calculated as the exponent of *β* and represented the odds of achieving higher utility.

Attribute part-worth (relative importance score) represents the average relative effect of each attribute on the change in the regimen’s utility and it is defined as the absolute difference between the highest and lowest coefficients in the range of preference weights within that attribute, divided by the total of the ranges of preference weights within all other attributes (including the price). In comparison to the other attributes in the model, it indicates the degree to which that attribute affects overall utility^18^. The WTP values represent the average amount of money in EGP the respondents are willing to pay to purchase the brand of each of the four CHD drug classes included in the DCE. Negative values mean the brand is less preferred than the generic for the corresponding class. To calculate the attribute partworths, we multiplied the preference weight of the price by 285 to account for the highest price presented to the respondents because the price is a continuous attribute. This adjustment estimates the change in utility between the best and worst prices.

We conducted four subgroup analyses based on sex, age, education, and income. For age, we split the sample of the dissatisfied respondents into two groups: above and below median age. We created two education groups: one with at least intermediate education and one below intermediate education. Finally, we split the sample into two income groups: one in debt and one at least meeting the routine expenses (not in debt).

R Software version 4.1.1.^19^ was used for data analysis and modeling. The mlogit package version 1.1-1^20^ was used to conduct conditional logistic regression.

### Ethical considerations

The study and all experimental protocols were approved by the HIO administration and the research and ethics committee of the central directorate for research and health development in the Egyptian Ministry of Health and Population. All methods were performed in accordance with relevant guidelines/regulations. An informed consent was obtained from all subjects and/or their legal guardian(s), after providing them with information about the study purpose.

## Results

During a three-month period, 208 CHD patients were surveyed from July to October 2021. The mean age was 67 ± 8 years, and 78% were males. Most of the patients (86%) were nonworking pensioners, and 114 (55%) had at least intermediate education. Seventy-six patients (37%) were in debt, while the remaining had incomes at least meeting their routine expenses. The initial screening of the respondents revealed that 59 patients (28%) were satisfied with all their generic medicines, while the other 149 patients were dissatisfied with at least one of their generic medicines; only the latter group was included in the discrete choice experiment. Table 1 summarizes the sociodemographic characteristics stratified by generic satisfaction status.

**Table 1:** Sociodemographic characteristics of the respondents stratified by their generic satisfaction status.

Characteristics	Satisfied (n = 59)	Dissatisfied (n = 149)	*(p* value)^a^
Mean age in years	68.8 ± 7.7	66.8 ± 8.2	(0.094)
Male (%)	49 (83.1%)	114 (76.5%)	(0.398)
Highest level of education			(0.020)*
Illiterate, read-&-write or primary	27 (45.8%)	43 (28.9%)	
Preparatory, secondary, graduate, or postgraduate degree	32 (54.2%)	106 (71.1%)	
Occupation			(1.000)
Not working (pensioners)	50 (84.7%)	128 (85.9%)	
Working	9 (15.3%)	21 (14.1%)	
Income level			(0.057)
In debt	14 (23.7%)	62 (41.6%)	
Just meet routine expenses	28 (47.5%)	51 (34.2%)	
Meet routine expenses and emergencies	17 (28.8%)	33 (22.1%)	
Able to save/invest money	0 (0%)	3 (2.0%)	

*p value < 0.05. ^a^p value for age derived from t test; for categorical variables from chi-square test except income level where the p value was derived by exact test with Montecarlo simulation.

The results of the 2980 choices made by the 149 dissatisfied patients in the DCE are summarized in Table 2. All preference weights of the brands were found to be significant. All five attributes had a statistically significant effect on achieving higher utility for CHD patients. The introduction of the branded versions of RAAS blockers, beta blockers and statins was associated with a significant increase in the odds of achieving higher utility. It is expected to increase by approximately three times (AOR 3.14; 95% CI 2.83 to 3.48) with the branded RAAS blocker and two times (AOR = 2.06 to 95% 1.88, 2.27) with the branded beta blocker. A 50% increase in the odds was observed with branded statins (AOR = 1.50, 95% CI 1.33 to 1.68). To have the branded versions of RAAS blockers, beta blockers and statins, patients are willing to pay EGP 128, 81 and 45, respectively.

**Table 2:** Brands’ preference weights, adjusted odds ratios for preferring each brand over its generic counterpart, and the willingness to pay for the brand.

Attribute	*β*	95% CI		AOR (95% CI)	WTP (95% CI)
		Lower	Upper		
Aspirin	− 0.338*	− 0.431	− 0.245	0.71 (0.65, 0.78)	EGP − 38 (− 73, − 16)
Beta-blocker	0.724*	0.630	0.818	2.06 (1.88, 2.27)	EGP 81 (53, 146)
Statin	0.403*	0.287	0.518	1.50 (1.33, 1.68)	EGP 45 (13, 63)
RAAS blocker	1.143*	1.039	1.247	3.14 (2.83, 3.48)	EGP 128 (93, 213)
Price^a^	− 0.450*	− 0.500	− 0.400	0.64 (0.61, 0.67)	
Highest	− 2.565^b^				

*AOR* adjusted odds ratio, *WTP* willingness to pay. ^a^Change in achieving higher utility associated with EGP 50 increase in the price, ^b^Refers to the preference weight (*β*) of the price multiplied by 285 to account for the highest price presented to the respondents. *p value < 0.001.

On the other hand, branded aspirin and higher prices were associated with a significant decrease in the odds of achieving higher utility, with a 29% reduction in the case of branded aspirin (AOR = 0.71; 95% CI 0.65 to 0.78) and a 36% reduction with every EGP 50 increase in price (AOR = 0.64; 95% CI 0.61 to 0.67).

RAAS blockers and beta blockers had the highest weights, followed by statins and aspirin (Table 2). The price weight was adjusted by multiplying by 285 to account for the highest price presented to the respondents. It was found to have the highest impact on utility with a preference weight of – 2.565. The WTP values in Table 2 were consistent with preference weights; positive values indicate higher preference toward the brand.

**Fig. 2:**
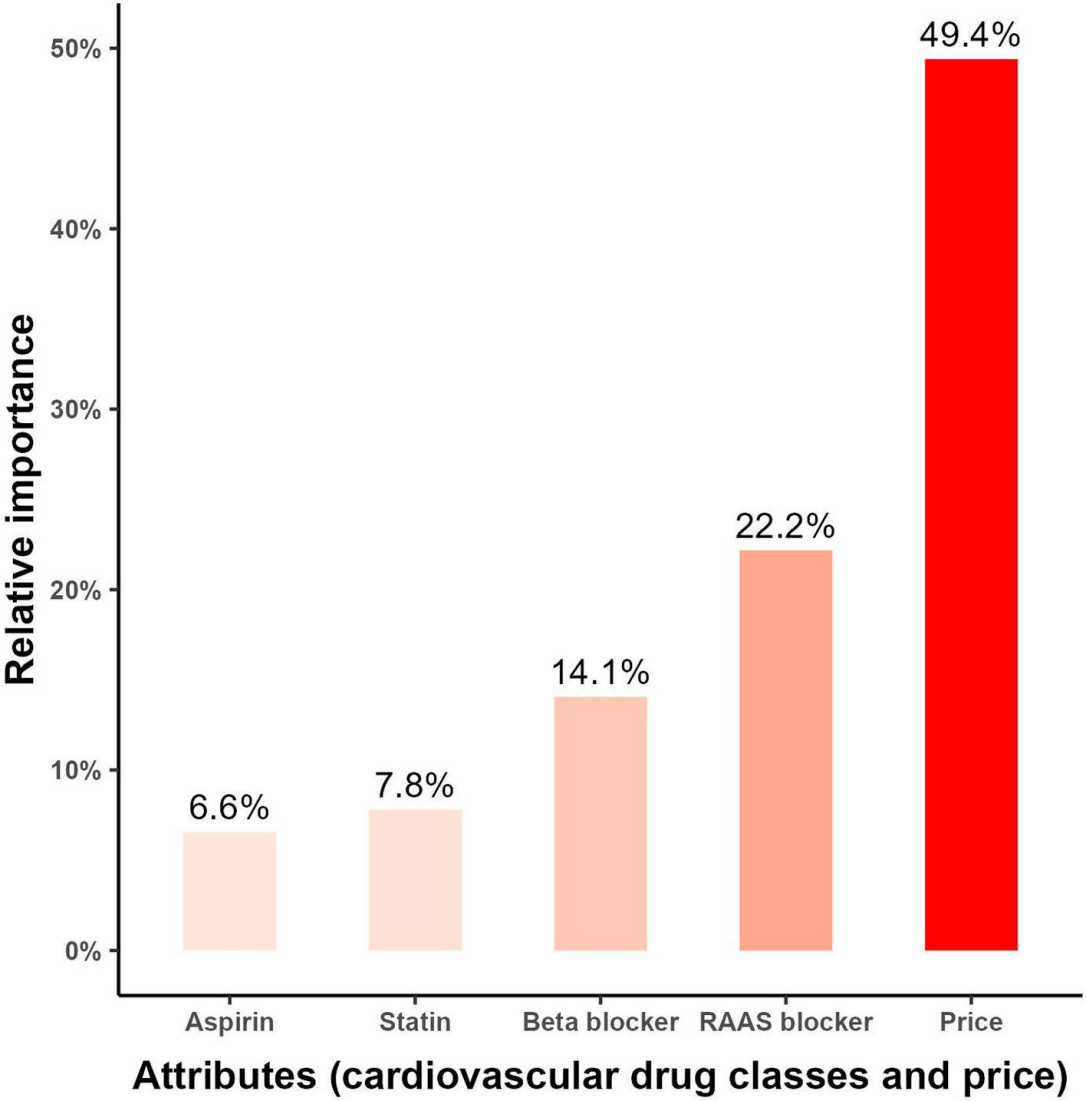
Relative importance of the four coronary heart disease (CHD) medications used for secondary prevention of CHD events from the patient perspective as revealed from the discrete choice experiment.

Figure 2 shows the relative importance of each class, pointing out that the RAAS blockers and beta-blockers had the highest importance from the patient’s perspective. In contrast, statins and aspirin had minor importance. The price had the highest relative importance at 49.4%.

### Subgroup analyses

In all subgroup analyses, we did not observe any changes in the direction of preferences (Appendices I, II, III, IV, and VIII). The subgroup analyses based on age showed that only price had higher importance in older age, while all drug classes had higher importance in younger age.

The relative importance of price and statins were higher among respondents with below intermediate education compared to those with at least intermediate education and among in debt respondents compared to those who at least meet routine expenses. Meanwhile, RAAS blockers, beta blockers and aspirin were more important among respondents with at least intermediate education and those at least meeting routine expenses.

### Drivers for brand inclinations

**Fig. 3:**
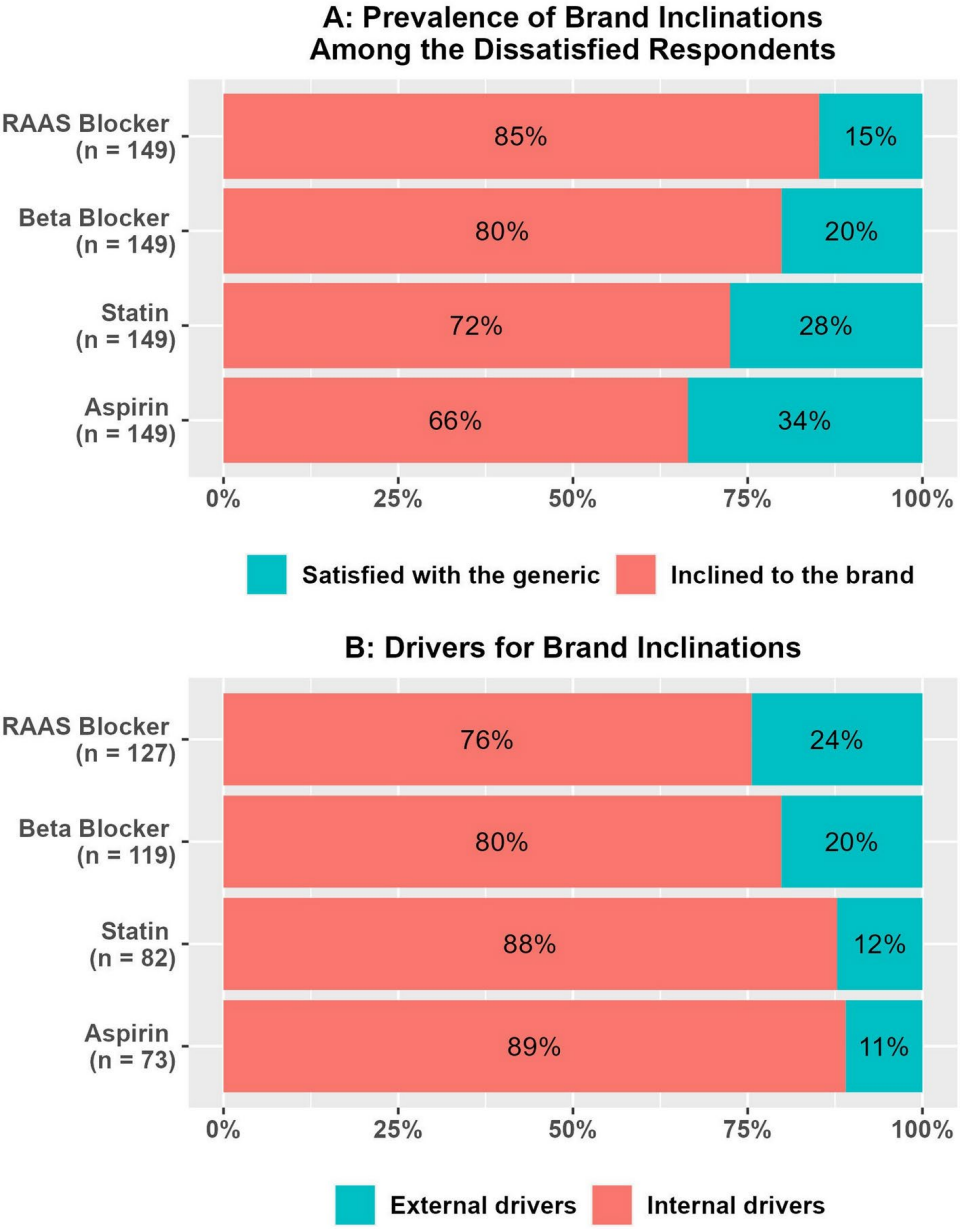
Prevalence of brand inclinations and their drivers for the four coronary heart disease (CHD) medications used for secondary prevention of CHD events.

For all four CHD medication classes, we found that most respondents were inclined to the brands (Fig. 3A); the percentages of those who had the intention to buy the brand and those who had already switched to the brand are provided in Appendix V. When those who had a brand inclination for each class were asked about what drove them to be inclined to the brand of that particular class, we found that most of the reasons were internally driven (Fig. 3B). Participants’ brand inclination drivers were categorized as internal drivers if they were related to their personal experiences using generic medications (ineffectiveness, adverse effects, or unfavorable perception about generic drugs in general), and external drivers if they were related to recommendations from others (health insurance doctors, private doctors, friends, or family). The main internal drivers were ineffectiveness (52.8% to 59.3%) and general negative perceptions about generics (22.8% to 35.4%). Side effects were minor drivers (Appendix VI).

## Discussion

This study demonstrated the usefulness of the DCE method to identify the brand-vs-generic patients’ preference weights for different classes of medicines used in the prevention of CHD. In our study, we found positive weights for the RAAS blockers, the beta-blockers, and the statins brands, which means more preference toward the brands for the three classes. In contrast, the aspirin brand was less preferred than the generic brand, as indicated by its negative weight; switching to the brand decreased the odds of having a high regimen utility by 29%. This finding contradicts the direct survey for aspirin, in which 66% of the respondents showed more inclination to the brand (Appendix V). However, when the respondents were faced with real marketplace simulation in DCE, the actual preferences were uncovered. This negative preference for the aspirin brand may be an indicator that Egyptian patients reject any drug change when they have no complaints.

We found that statins came third in the three classes with positive preference weights; the switch from its generic version to the brand had a 50% increase in the odds of having a high regimen utility, while RAAS blockers and beta-blockers had 214% and 106% increases, respectively. This marked difference with statins might be attributed to the fact that patients cannot easily notice and monitor their beneficial effects. For the classes used to control the heart rate or blood pressure, the patients can notice insufficient control by frequent monitoring of pulse and blood pressure available at home or community pharmacies. At the same time, there is no frequent monitoring of the total lipid profile performed at the clinic to provide evidence of insufficient lowering of LDL-cholesterol (if any) with statins.

In addition, the DCE helped us quantify each class’s relative importance from the patient’s perspectives and uncovered areas for improvements related to the patient’s understanding of the critical role of their CHD medications. Statins and aspirin had relatively low importance (8% and 7%, respectively) compared with the RAAS blockers and beta-blockers (22% and 14%, respectively). This finding highlights the need for intensive patient counseling to explain the crucial role of those classes in reducing mortality rates among CHD patients. We need to stress monitoring the lipid profiles and check the need for any required dose modifications.

When we examined the WTP for the brands of the three classes with positive brand weights, we found that the respondents, on average, had higher WTP than the actual average prices for the brands available through the HIO pharmacies for RAAS blockers (EGP 128 vs EGP 68) and beta-blockers (EGP 81 vs EGP 41). In contrast, the WTP for the statins’ brands was below their actual average prices (EGP 45 vs EGP 154). The rankings of the WTP values for the classes’ brands are another reflection of their relative importance; the substantially low WTP for the statins’ brands illustrates how the patients perceived their importance.

The preference weights for the brands of all classes contained in a standard treatment regimen used for one medical condition were measured in our investigation, the first discrete choice experiment to do so (as far as we are aware). We only found one study, by Halme et al., that used the DCE to examine consumers’ preferences for one branded painkiller and its theoretical, generic alternatives; however, the suggested generics may not be bioequivalent to the brand because of differences in the proposed generics’ features from the brand^13^.

The preferences for branded and generic medications in a specific group with serious diseases were the subject of our study, one of the few studies to do so. Other studies with similar aims asked questions about just one class of medication that the population being studied was taking, such as generic antiepileptics^5,6^, generic antiretrovirals^7,8^, generic cyclosporin^9^, and atypical antipsychotics^10^. None of those studies measured the relative preference weights of different classes included in one regimen, as in our study.

Among the studies that examined the effect of the seriousness of different conditions on the preferences of patients toward the generics and brands, we identified one study that employed a nine-point semantic difference scale to provide indirect weights for the brands in the form of the level of risk associated with using the generic for various medical conditions and the necessary cost savings amounts to accepting the generic substitution^11^. The differences in the risk scores for the included medical conditions were tested by repeated measures MANOVA. However, our DCE approach had three unique advantages over the 9-point semantic scale. First, it jointly extracts relative preference weights in a simulation of an actual marketplace that the patients easily interpret. Second, it provides relative importance scores for each class, giving insights into potential patient counseling areas. Third, it provides WTP values in a better way than contingent valuation methods that are usually affected by the limited respondent’s ability to pay.

The Alexandria Health Insurance Clinic sees a large number of patients every day, and it serves a sizable number of beneficiaries in Alexandria (8.4% of the city’s population), which makes the study’s setting one of its strengths, as it represents the system of health insurance clinics applied in Egypt. Additionally, the context assisted us in overcoming the low level of generic medicine awareness among Egyptian patients who Elmoneer discovered in her unpublished study when she evaluated the knowledge, perceptions, and acceptability of generic substitution in Egypt^21^. The clinic’s pharmacy was divided into two sections: one for free medications with three dispensing windows and the other with one window for brand-name medications. This context helped us define generics as those medications offered without copayments (referred to by patients as insurance medications or "Dawa El Taamin" in Arabic) and brands as those medications for which a patient pays the difference and receives them from the brands’ outlet. We found our patients familiar with those definitions. If we had done the trial in a private drugstore, we would have had trouble getting the patients to understand the difference between brand-name and generic medications.

This study has some limitations. First, the findings regarding Egyptian patients cannot be generalized to patients in other countries with different health systems, quality of generic drugs, and patient awareness. Second, this study was limited to the four CVD medicine classes indicated for secondary prevention of ASCVD and are all shared by the respondents, we could not include P2Y12 inhibitors, aldosterone antagonists, trimetazidine, or CCBs in the DCE due to the relatively small number of surveyed patients who use such medications. However, other researchers might choose a larger sample from different centers to study any given number of CHD medicines. Third, this study did not investigate the direct effect of brand inclination on medication adherence nor the impact of factors associated with brand preference on medication adherence. We recommend future studies address this point and compare the medication adherence between patients who choose to pay for the brands and those who do not afford to pay and are obliged to use the generic versions. Fourth, while this study assumes the bioequivalence of generic medicines to their branded counterparts in accordance with Egyptian pharmaceutical regulations, we acknowledge a significant limitation in that there is an absence of published evidence confirming such bioequivalence. Although regulatory standards are met, the actual verification through direct analysis of drug content or blind testing among patients was beyond the scope and resources of this study. We recommend that future research should include experimental methods to directly confirm the quality and effectiveness of generic medications, thereby strengthening the evidence base for bioequivalence.

The results of our study call for future research to ascertain the causes of the brand inclinations that were primarily patient-driven in the four CHD classes despite the regulatory bioequivalence requirements imposed by the Egyptian drug authority on the generic drug manufacturers to grant marketing approval of generic products. According to these regulations, drug manufacturers must demonstrate that their generic pharmaceutical products meet the same quality, safety, and efficacy standards as the innovator or reference listed product(s) and that, when administered at the same molar dose of the therapeutic ingredient(s) under similar experimental conditions, either a single dose or multiple doses, the drug’s rate and extent of absorption do not significantly differ from the reference listed product’s rate and extent of absorption^22^. We advise undertaking cohort studies to compare the clinical efficacy of the brands with their generics to ensure health equity for all patients. These studies are essential to making the right decisions, such as whether to revise the bioequivalence testing of the previously approved generics currently on the market or use patient counseling to reassure patients at the Alexandria clinic and other health insurance clinics about the quality of generic medications. The primary strength of this study lies in the novel application of the DCE methodology to the field of studying brand-versus-generic drug preferences. This methodology aims to reveal patients’ perspectives regarding the relative importance of drugs from a fixed regimen of pharmacological classes in determining the regimen’s utility for the patients. Such insights could help clinicians correct patients’ misconceptions or address their lack of sufficient drug information.

## Conclusions

In a cohort of CHD patients covered by health insurance in Alexandria, we found a high brand inclination for three of the four medications recommended for secondary prevention of CHD. The relative importance of aspirin and statins were low compared to RAAS blockers and Beta-blockers. These findings have therapeutic implications for the HIO clinics’ clinical practices. Firstly, in order to ensure patient satisfaction and medication adherence, physicians and pharmacists must reassure patients about the quality of generic medications. Secondly, observational studies are required by health insurance providers to verify the effectiveness of generic CHD medications. Finally, the fact that aspirin and statins had minor relative importance from the patients’ perspectives, despite their well-established preventive benefits, emphasizes the necessity of increasing patient awareness. Our study added evidence supporting the utility of the DCE method to compare the preferences of branded and generic drugs used in CHD prevention.

## Supplementary Information

Supplementary Information 1.

Supplementary Information 2.

Supplementary Information 3.

Supplementary Information 4.

## Data Availability

The datasets used and/or analyzed during the current study are available from the corresponding author on reasonable request.

## Abbreviations

ACS: Acute coronary syndrome
AOR: Adjusted odds ratio
CHD: Coronary heart disease
DCE: Discrete choice experiment
LDL: Low-density lipoprotein
RAAS blockers: Renin–angiotensin–aldosterone system blockers
HIO: Health insurance organization
ISPOR: International Society of Pharmacoeconomics and Outcomes Research
WTP: Willingness to pay
