# Supplementary material for: Uncovering patients’ preferences for brand among essential classes of coronary heart disease medications using a discrete choice experiment
Source: Sci Rep. 2024 Nov 4;14:26643. doi: 10.1038/s41598-024-77007-3 (PMC11535387; doi:10.1038/s41598-024-77007-3)
Supplement: Supplementary file 1 — Supplementary Information 1. [file 41598_2024_77007_MOESM1_ESM.pdf]

BLOCK 1

CHOICE SET 1

Attribute	Alternative 1	Alternative 2	Status Quo
Aspirin	Brand	Generic	
Beta Blocker	Brand	Brand	
Statin	Brand	Generic	
RAAS Blocker	Brand	Generic	
Price	285	41	
Adapted Price			

CHOICE SET 2

Attribute	Alternative 1	Alternative 2	Status Quo
Aspirin	Generic	Brand	
Beta Blocker	Generic	Brand	
Statin	Brand	Generic	
RAAS Blocker	Brand	Brand	
Price	222	131	
Adapted Price			

CHOICE SET 3

Attribute	Alternative 1	Alternative 2	Status Quo
Aspirin	Generic	Brand	
Beta Blocker	Brand	Generic	
Statin	Brand	Generic	
RAAS Blocker	Generic	Generic	
Price	195	22	
Adapted Price			

CHOICE SET 4

Attribute	Alternative 1	Alternative 2	Status Quo
Aspirin	Brand	Generic	
Beta Blocker	Brand	Generic	
Statin	Brand	Brand	
RAAS Blocker	Brand	Generic	
Price	285	154	
Adapted Price			

CHOICE SET 5

Attribute	Alternative 1	Alternative 2	Status Quo
Aspirin	Generic	Brand	
Beta Blocker	Brand	Generic	
Statin	Brand	Generic	
RAAS Blocker	Brand	Generic	
Price	263	22	
Adapted Price			

CHOICE SET 6

Attribute	Alternative 1	Alternative 2	Status Quo
Aspirin	Brand	Generic	
Beta Blocker	Generic	Brand	
Statin	Brand	Generic	
RAAS Blocker	Brand	Generic	
Price	244	41	
Adapted Price			

CHOICE SET 7

Attribute	Alternative 1	Alternative 2	Status Quo
Aspirin	Brand	Generic	
Beta Blocker	Brand	Generic	
Statin	Brand	Generic	
RAAS Blocker	Brand	Brand	
Price	285	68	
Adapted Price			

CHOICE SET 8

Attribute	Alternative 1	Alternative 2	Status Quo
Aspirin	Brand	Generic	
Beta Blocker	Brand	Generic	
Statin	Generic	Brand	
RAAS Blocker	Brand	Generic	
Price	131	154	
Adapted Price			

CHOICE SET 9

Attribute	Alternative 1	Alternative 2	Status Quo
Aspirin	Brand	Brand	
Beta Blocker	Generic	Generic	
Statin	Generic	Brand	
RAAS Blocker	Brand	Generic	
Price	90	176	
Adapted Price			

CHOICE SET 10

Attribute	Alternative 1	Alternative 2	Status Quo
Aspirin	Brand	Generic	
Beta Blocker	Brand	Generic	
Statin	Generic	Generic	
RAAS Blocker	Brand	Generic	
Price	131	0	
Adapted Price			

BLOCK 2

CHOICE SET 1

Attribute	Alternative 1	Alternative 2	Status Quo
Aspirin	Generic	Brand	
Beta Blocker	Generic	Brand	
Statin	Brand	Generic	
RAAS Blocker	Brand	Generic	
Price	222	63	
Adapted Price			

CHOICE SET 2

Attribute	Alternative 1	Alternative 2	Status Quo
Aspirin	Generic	Brand	
Beta Blocker	Generic	Generic	
Statin	Brand	Generic	
RAAS Blocker	Brand	Generic	
Price	222	22	
Adapted Price			

CHOICE SET 3

Attribute	Alternative 1	Alternative 2	Status Quo
Aspirin	Generic	Generic	
Beta Blocker	Brand	Generic	
Statin	Brand	Generic	
RAAS Blocker	Brand	Generic	
Price	263	0	
Adapted Price			

CHOICE SET 4

Attribute	Alternative 1	Alternative 2	Status Quo
Aspirin	Generic	Brand	
Beta Blocker	Brand	Generic	
Statin	Generic	Generic	
RAAS Blocker	Brand	Generic	
Price	109	22	
Adapted Price			

CHOICE SET 5

Attribute	Alternative 1	Alternative 2	Status Quo
Aspirin	Brand	Generic	
Beta Blocker	Generic	Brand	
Statin	Generic	Brand	
RAAS Blocker	Brand	Generic	
Price	90	195	
Adapted Price			

CHOICE SET 6

Attribute	Alternative 1	Alternative 2	Status Quo
Aspirin	Generic	Generic	
Beta Blocker	Generic	Brand	
Statin	Generic	Brand	
RAAS Blocker	Brand	Generic	
Price	68	195	
Adapted Price			

CHOICE SET 7

Attribute	Alternative 1	Alternative 2	Status Quo
Aspirin	Brand	Generic	
Beta Blocker	Generic	Brand	
Statin	Brand	Generic	
RAAS Blocker	Generic	Generic	
Price	176	41	
Adapted Price			

CHOICE SET 8

Attribute	Alternative 1	Alternative 2	Status Quo
Aspirin	Generic	Brand	
Beta Blocker	Brand	Generic	
Statin	Generic	Brand	
RAAS Blocker	Brand	Generic	
Price	109	176	
Adapted Price			

CHOICE SET 9

Attribute	Alternative 1	Alternative 2	Status Quo
Aspirin	Brand	Brand	
Beta Blocker	Brand	Generic	
Statin	Brand	Generic	
RAAS Blocker	Brand	Generic	
Price	285	22	
Adapted Price			

CHOICE SET 10

Attribute	Alternative 1	Alternative 2	Status Quo
Aspirin	Generic	Brand	
Beta Blocker	Brand	Brand	
Statin	Brand	Brand	
RAAS Blocker	Brand	Generic	
Price	263	217	
Adapted Price			

BLOCK 3

CHOICE SET 1

Attribute	Alternative 1	Alternative 2	Status Quo
Aspirin	Brand	Brand	
Beta Blocker	Brand	Generic	
Statin	Brand	Generic	
RAAS Blocker	Brand	Generic	
Price	285	22	
Adapted Price			

CHOICE SET 2

Attribute	Alternative 1	Alternative 2	Status Quo
Aspirin	Generic	Brand	
Beta Blocker	Generic	Brand	
Statin	Brand	Generic	
RAAS Blocker	Brand	Brand	
Price	222	131	
Adapted Price			

CHOICE SET 3

Attribute	Alternative 1	Alternative 2	Status Quo
Aspirin	Generic	Brand	
Beta Blocker	Brand	Generic	
Statin	Brand	Generic	
RAAS Blocker	Generic	Generic	
Price	195	22	
Adapted Price			

CHOICE SET 4

Attribute	Alternative 1	Alternative 2	Status Quo
Aspirin	Brand	Generic	
Beta Blocker	Generic	Brand	
Statin	Brand	Generic	
RAAS Blocker	Brand	Generic	
Price	244	41	
Adapted Price			

CHOICE SET 5

Attribute	Alternative 1	Alternative 2	Status Quo
Aspirin	Generic	Generic	
Beta Blocker	Brand	Generic	
Statin	Brand	Generic	
RAAS Blocker	Brand	Generic	
Price	263	0	
Adapted Price			

CHOICE SET 6

Attribute	Alternative 1	Alternative 2	Status Quo
Aspirin	Generic	Brand	
Beta Blocker	Brand	Generic	
Statin	Generic	Brand	
RAAS Blocker	Brand	Generic	
Price	109	176	
Adapted Price			

CHOICE SET 7

Attribute	Alternative 1	Alternative 2	Status Quo
Aspirin	Brand	Generic	
Beta Blocker	Brand	Generic	
Statin	Brand	Brand	
RAAS Blocker	Brand	Generic	
Price	285	154	
Adapted Price			

CHOICE SET 8

Attribute	Alternative 1	Alternative 2	Status Quo
Aspirin	Brand	Generic	
Beta Blocker	Brand	Generic	
Statin	Generic	Brand	
RAAS Blocker	Brand	Generic	
Price	131	154	
Adapted Price			

CHOICE SET 9

Attribute	Alternative 1	Alternative 2	Status Quo
Aspirin	Brand	Generic	
Beta Blocker	Generic	Brand	
Statin	Generic	Brand	
RAAS Blocker	Brand	Generic	
Price	90	195	
Adapted Price			

CHOICE SET 10

Attribute	Alternative 1	Alternative 2	Status Quo
Aspirin	Generic	Brand	
Beta Blocker	Brand	Generic	
Statin	Brand	Generic	
RAAS Blocker	Brand	Generic	
Price	263	22	
Adapted Price			

BLOCK 4

CHOICE SET 1

Attribute	Alternative 1	Alternative 2	Status Quo
Aspirin	Brand	Generic	
Beta Blocker	Brand	Brand	
Statin	Brand	Generic	
RAAS Blocker	Brand	Generic	
Price	285	41	
Adapted Price			

CHOICE SET 6

Attribute	Alternative 1	Alternative 2	Status Quo
Aspirin	Brand	Brand	
Beta Blocker	Generic	Generic	
Statin	Generic	Brand	
RAAS Blocker	Brand	Generic	
Price	90	176	
Adapted Price			

CHOICE SET 2

Attribute	Alternative 1	Alternative 2	Status Quo
Aspirin	Generic	Brand	
Beta Blocker	Generic	Generic	
Statin	Brand	Generic	
RAAS Blocker	Brand	Generic	
Price	222	22	
Adapted Price			

CHOICE SET 7

Attribute	Alternative 1	Alternative 2	Status Quo
Aspirin	Brand	Generic	
Beta Blocker	Brand	Generic	
Statin	Brand	Generic	
RAAS Blocker	Brand	Brand	
Price	285	68	
Adapted Price			

CHOICE SET 3

Attribute	Alternative 1	Alternative 2	Status Quo
Aspirin	Generic	Brand	
Beta Blocker	Brand	Generic	
Statin	Generic	Generic	
RAAS Blocker	Brand	Generic	
Price	109	22	
Adapted Price			

CHOICE SET 8

Attribute	Alternative 1	Alternative 2	Status Quo
Aspirin	Brand	Generic	
Beta Blocker	Brand	Generic	
Statin	Generic	Generic	
RAAS Blocker	Brand	Generic	
Price	131	0	
Adapted Price			

CHOICE SET 4

Attribute	Alternative 1	Alternative 2	Status Quo
Aspirin	Generic	Brand	
Beta Blocker	Generic	Brand	
Statin	Brand	Generic	
RAAS Blocker	Brand	Generic	
Price	222	63	
Adapted Price			

CHOICE SET 9

Attribute	Alternative 1	Alternative 2	Status Quo
Aspirin	Brand	Generic	
Beta Blocker	Generic	Brand	
Statin	Brand	Generic	
RAAS Blocker	Generic	Generic	
Price	176	41	
Adapted Price			

CHOICE SET 5

Attribute	Alternative 1	Alternative 2	Status Quo
Aspirin	Generic	Generic	
Beta Blocker	Generic	Brand	
Statin	Generic	Brand	
RAAS Blocker	Brand	Generic	
Price	68	195	
Adapted Price			

CHOICE SET 10

Attribute	Alternative 1	Alternative 2	Status Quo
Aspirin	Generic	Brand	
Beta Blocker	Brand	Brand	
Statin	Brand	Brand	
RAAS Blocker	Brand	Generic	
Price	263	217	
Adapted Price			

BLOCK 5

CHOICE SET 1

Attribute	Alternative 1	Alternative 2	Status Quo
Aspirin	Brand	Generic	
Beta Blocker	Generic	Brand	
Statin	Brand	Generic	
RAAS Blocker	Brand	Generic	
Price	244	41	
Adapted Price			

CHOICE SET 2

Attribute	Alternative 1	Alternative 2	Status Quo
Aspirin	Brand	Generic	
Beta Blocker	Brand	Brand	
Statin	Brand	Generic	
RAAS Blocker	Brand	Generic	
Price	285	41	
Adapted Price			

CHOICE SET 3

Attribute	Alternative 1	Alternative 2	Status Quo
Aspirin	Generic	Brand	
Beta Blocker	Brand	Brand	
Statin	Brand	Brand	
RAAS Blocker	Brand	Generic	
Price	263	217	
Adapted Price			

CHOICE SET 4

Attribute	Alternative 1	Alternative 2	Status Quo
Aspirin	Generic	Brand	
Beta Blocker	Generic	Brand	
Statin	Brand	Generic	
RAAS Blocker	Brand	Brand	
Price	222	131	
Adapted Price			

CHOICE SET 5

Attribute	Alternative 1	Alternative 2	Status Quo
Aspirin	Brand	Brand	
Beta Blocker	Generic	Generic	
Statin	Generic	Brand	
RAAS Blocker	Brand	Generic	
Price	90	176	
Adapted Price			

CHOICE SET 6

Attribute	Alternative 1	Alternative 2	Status Quo
Aspirin	Brand	Generic	
Beta Blocker	Generic	Brand	
Statin	Generic	Brand	
RAAS Blocker	Brand	Generic	
Price	90	195	
Adapted Price			

CHOICE SET 7

Attribute	Alternative 1	Alternative 2	Status Quo
Aspirin	Generic	Generic	
Beta Blocker	Brand	Generic	
Statin	Brand	Generic	
RAAS Blocker	Brand	Generic	
Price	263	0	
Adapted Price			

CHOICE SET 8

Attribute	Alternative 1	Alternative 2	Status Quo
Aspirin	Brand	Generic	
Beta Blocker	Generic	Brand	
Statin	Brand	Generic	
RAAS Blocker	Generic	Generic	
Price	176	41	
Adapted Price			

CHOICE SET 9

Attribute	Alternative 1	Alternative 2	Status Quo
Aspirin	Generic	Brand	
Beta Blocker	Generic	Brand	
Statin	Brand	Generic	
RAAS Blocker	Brand	Generic	
Price	222	63	
Adapted Price			

CHOICE SET 10

Attribute	Alternative 1	Alternative 2	Status Quo
Aspirin	Brand	Generic	
Beta Blocker	Brand	Generic	
Statin	Generic	Generic	
RAAS Blocker	Brand	Generic	
Price	131	0	
Adapted Price			

BLOCK 6

CHOICE SET 1

Attribute	Alternative 1	Alternative 2	Status Quo
Aspirin	Generic	Generic	
Beta Blocker	Generic	Brand	
Statin	Generic	Brand	
RAAS Blocker	Brand	Generic	
Price	68	195	
Adapted Price			

CHOICE SET 2

Attribute	Alternative 1	Alternative 2	Status Quo
Aspirin	Brand	Generic	
Beta Blocker	Brand	Generic	
Statin	Brand	Brand	
RAAS Blocker	Brand	Generic	
Price	285	154	
Adapted Price			

CHOICE SET 3

Attribute	Alternative 1	Alternative 2	Status Quo
Aspirin	Generic	Brand	
Beta Blocker	Brand	Generic	
Statin	Generic	Generic	
RAAS Blocker	Brand	Generic	
Price	109	22	
Adapted Price			

CHOICE SET 4

Attribute	Alternative 1	Alternative 2	Status Quo
Aspirin	Generic	Brand	
Beta Blocker	Brand	Generic	
Statin	Brand	Generic	
RAAS Blocker	Brand	Generic	
Price	263	22	
Adapted Price			

CHOICE SET 5

Attribute	Alternative 1	Alternative 2	Status Quo
Aspirin	Generic	Brand	
Beta Blocker	Brand	Generic	
Statin	Brand	Generic	
RAAS Blocker	Generic	Generic	
Price	195	22	
Adapted Price			

CHOICE SET 6

Attribute	Alternative 1	Alternative 2	Status Quo
Aspirin	Brand	Brand	
Beta Blocker	Brand	Generic	
Statin	Brand	Generic	
RAAS Blocker	Brand	Generic	
Price	285	22	
Adapted Price			

CHOICE SET 7

Attribute	Alternative 1	Alternative 2	Status Quo
Aspirin	Generic	Brand	
Beta Blocker	Generic	Generic	
Statin	Brand	Generic	
RAAS Blocker	Brand	Generic	
Price	222	22	
Adapted Price			

CHOICE SET 8

Attribute	Alternative 1	Alternative 2	Status Quo
Aspirin	Brand	Generic	
Beta Blocker	Brand	Generic	
Statin	Brand	Generic	
RAAS Blocker	Brand	Brand	
Price	285	68	
Adapted Price			

CHOICE SET 9

Attribute	Alternative 1	Alternative 2	Status Quo
Aspirin	Generic	Brand	
Beta Blocker	Brand	Generic	
Statin	Generic	Brand	
RAAS Blocker	Brand	Generic	
Price	109	176	
Adapted Price			

CHOICE SET 10

Attribute	Alternative 1	Alternative 2	Status Quo
Aspirin	Brand	Generic	
Beta Blocker	Brand	Generic	
Statin	Generic	Brand	
RAAS Blocker	Brand	Generic	
Price	131	154	
Adapted Price			

BLOCK 7

CHOICE SET 1

Attribute	Alternative 1	Alternative 2	Status Quo
Aspirin	Generic	Brand	
Beta Blocker	Generic	Generic	
Statin	Brand	Generic	
RAAS Blocker	Brand	Generic	
Price	222	22	
Adapted Price			

CHOICE SET 2

Attribute	Alternative 1	Alternative 2	Status Quo
Aspirin	Generic	Brand	
Beta Blocker	Brand	Generic	
Statin	Brand	Generic	
RAAS Blocker	Brand	Generic	
Price	263	22	
Adapted Price			

CHOICE SET 3

Attribute	Alternative 1	Alternative 2	Status Quo
Aspirin	Generic	Generic	
Beta Blocker	Brand	Generic	
Statin	Brand	Generic	
RAAS Blocker	Brand	Generic	
Price	263	0	
Adapted Price			

CHOICE SET 4

Attribute	Alternative 1	Alternative 2	Status Quo
Aspirin	Generic	Generic	
Beta Blocker	Generic	Brand	
Statin	Generic	Brand	
RAAS Blocker	Brand	Generic	
Price	68	195	
Adapted Price			

CHOICE SET 5

Attribute	Alternative 1	Alternative 2	Status Quo
Aspirin	Generic	Brand	
Beta Blocker	Brand	Generic	
Statin	Generic	Brand	
RAAS Blocker	Brand	Generic	
Price	109	176	
Adapted Price			

CHOICE SET 6

Attribute	Alternative 1	Alternative 2	Status Quo
Aspirin	Generic	Brand	
Beta Blocker	Brand	Generic	
Statin	Brand	Generic	
RAAS Blocker	Generic	Generic	
Price	195	22	
Adapted Price			

CHOICE SET 7

Attribute	Alternative 1	Alternative 2	Status Quo
Aspirin	Brand	Brand	
Beta Blocker	Brand	Generic	
Statin	Brand	Generic	
RAAS Blocker	Brand	Generic	
Price	285	22	
Adapted Price			

CHOICE SET 8

Attribute	Alternative 1	Alternative 2	Status Quo
Aspirin	Brand	Generic	
Beta Blocker	Brand	Generic	
Statin	Generic	Generic	
RAAS Blocker	Brand	Generic	
Price	131	0	
Adapted Price			

CHOICE SET 9

Attribute	Alternative 1	Alternative 2	Status Quo
Aspirin	Generic	Brand	
Beta Blocker	Generic	Brand	
Statin	Brand	Generic	
RAAS Blocker	Brand	Brand	
Price	222	131	
Adapted Price			

CHOICE SET 10

Attribute	Alternative 1	Alternative 2	Status Quo
Aspirin	Brand	Generic	
Beta Blocker	Brand	Brand	
Statin	Brand	Generic	
RAAS Blocker	Brand	Generic	
Price	285	41	
Adapted Price			

BLOCK 8

CHOICE SET 1

Attribute	Alternative 1	Alternative 2	Status Quo
Aspirin	Generic	Brand	
Beta Blocker	Brand	Brand	
Statin	Brand	Brand	
RAAS Blocker	Brand	Generic	
Price	263	217	
Adapted Price			

CHOICE SET 2

Attribute	Alternative 1	Alternative 2	Status Quo
Aspirin	Brand	Brand	
Beta Blocker	Generic	Generic	
Statin	Generic	Brand	
RAAS Blocker	Brand	Generic	
Price	90	176	
Adapted Price			

CHOICE SET 3

Attribute	Alternative 1	Alternative 2	Status Quo
Aspirin	Brand	Generic	
Beta Blocker	Generic	Brand	
Statin	Brand	Generic	
RAAS Blocker	Brand	Generic	
Price	244	41	
Adapted Price			

CHOICE SET 4

Attribute	Alternative 1	Alternative 2	Status Quo
Aspirin	Generic	Brand	
Beta Blocker	Brand	Generic	
Statin	Generic	Generic	
RAAS Blocker	Brand	Generic	
Price	109	22	
Adapted Price			

CHOICE SET 5

Attribute	Alternative 1	Alternative 2	Status Quo
Aspirin	Brand	Generic	
Beta Blocker	Brand	Generic	
Statin	Generic	Brand	
RAAS Blocker	Brand	Generic	
Price	131	154	
Adapted Price			

CHOICE SET 6

Attribute	Alternative 1	Alternative 2	Status Quo
Aspirin	Generic	Brand	
Beta Blocker	Generic	Brand	
Statin	Brand	Generic	
RAAS Blocker	Brand	Generic	
Price	222	63	
Adapted Price			

CHOICE SET 7

Attribute	Alternative 1	Alternative 2	Status Quo
Aspirin	Brand	Generic	
Beta Blocker	Brand	Generic	
Statin	Brand	Generic	
RAAS Blocker	Brand	Brand	
Price	285	68	
Adapted Price			

CHOICE SET 8

Attribute	Alternative 1	Alternative 2	Status Quo
Aspirin	Brand	Generic	
Beta Blocker	Generic	Brand	
Statin	Brand	Generic	
RAAS Blocker	Generic	Generic	
Price	176	41	
Adapted Price			

CHOICE SET 9

Attribute	Alternative 1	Alternative 2	Status Quo
Aspirin	Brand	Generic	
Beta Blocker	Generic	Brand	
Statin	Generic	Brand	
RAAS Blocker	Brand	Generic	
Price	90	195	
Adapted Price			

CHOICE SET 10

Attribute	Alternative 1	Alternative 2	Status Quo
Aspirin	Brand	Generic	
Beta Blocker	Brand	Generic	
Statin	Brand	Brand	
RAAS Blocker	Brand	Generic	
Price	285	154	
Adapted Price			

BLOCK 9

CHOICE SET 1

Attribute	Alternative 1	Alternative 2	Status Quo
Aspirin	Brand	Generic	
Beta Blocker	Brand	Generic	
Statin	Generic	Brand	
RAAS Blocker	Brand	Generic	
Price	131	154	
Adapted Price			

CHOICE SET 2

Attribute	Alternative 1	Alternative 2	Status Quo
Aspirin	Generic	Brand	
Beta Blocker	Brand	Brand	
Statin	Brand	Brand	
RAAS Blocker	Brand	Generic	
Price	263	217	
Adapted Price			

CHOICE SET 3

Attribute	Alternative 1	Alternative 2	Status Quo
Aspirin	Generic	Brand	
Beta Blocker	Generic	Brand	
Statin	Brand	Generic	
RAAS Blocker	Brand	Brand	
Price	222	131	
Adapted Price			

CHOICE SET 4

Attribute	Alternative 1	Alternative 2	Status Quo
Aspirin	Generic	Brand	
Beta Blocker	Generic	Generic	
Statin	Brand	Generic	
RAAS Blocker	Brand	Generic	
Price	222	22	
Adapted Price			

CHOICE SET 5

Attribute	Alternative 1	Alternative 2	Status Quo
Aspirin	Brand	Generic	
Beta Blocker	Brand	Generic	
Statin	Generic	Generic	
RAAS Blocker	Brand	Generic	
Price	131	0	
Adapted Price			

CHOICE SET 6

Attribute	Alternative 1	Alternative 2	Status Quo
Aspirin	Brand	Brand	
Beta Blocker	Brand	Generic	
Statin	Brand	Generic	
RAAS Blocker	Brand	Generic	
Price	285	22	
Adapted Price			

CHOICE SET 7

Attribute	Alternative 1	Alternative 2	Status Quo
Aspirin	Brand	Generic	
Beta Blocker	Generic	Brand	
Statin	Brand	Generic	
RAAS Blocker	Brand	Generic	
Price	244	41	
Adapted Price			

CHOICE SET 8

Attribute	Alternative 1	Alternative 2	Status Quo
Aspirin	Brand	Generic	
Beta Blocker	Brand	Generic	
Statin	Brand	Generic	
RAAS Blocker	Brand	Brand	
Price	285	68	
Adapted Price			

CHOICE SET 9

Attribute	Alternative 1	Alternative 2	Status Quo
Aspirin	Generic	Generic	
Beta Blocker	Brand	Generic	
Statin	Brand	Generic	
RAAS Blocker	Brand	Generic	
Price	263	0	
Adapted Price			

CHOICE SET 10

Attribute	Alternative 1	Alternative 2	Status Quo
Aspirin	Brand	Generic	
Beta Blocker	Generic	Brand	
Statin	Brand	Generic	
RAAS Blocker	Generic	Generic	
Price	176	41	
Adapted Price			

BLOCK 10

CHOICE SET 1

Attribute	Alternative 1	Alternative 2	Status Quo
Aspirin	Generic	Brand	
Beta Blocker	Brand	Generic	
Statin	Brand	Generic	
RAAS Blocker	Brand	Generic	
Price	263	22	
Adapted Price			

CHOICE SET 2

Attribute	Alternative 1	Alternative 2	Status Quo
Aspirin	Generic	Brand	
Beta Blocker	Brand	Generic	
Statin	Generic	Brand	
RAAS Blocker	Brand	Generic	
Price	109	176	
Adapted Price			

CHOICE SET 3

Attribute	Alternative 1	Alternative 2	Status Quo
Aspirin	Brand	Generic	
Beta Blocker	Generic	Brand	
Statin	Generic	Brand	
RAAS Blocker	Brand	Generic	
Price	90	195	
Adapted Price			

CHOICE SET 4

Attribute	Alternative 1	Alternative 2	Status Quo
Aspirin	Brand	Generic	
Beta Blocker	Brand	Brand	
Statin	Brand	Generic	
RAAS Blocker	Brand	Generic	
Price	285	41	
Adapted Price			

CHOICE SET 5

Attribute	Alternative 1	Alternative 2	Status Quo
Aspirin	Brand	Brand	
Beta Blocker	Generic	Generic	
Statin	Generic	Brand	
RAAS Blocker	Brand	Generic	
Price	90	176	
Adapted Price			

CHOICE SET 6

Attribute	Alternative 1	Alternative 2	Status Quo
Aspirin	Brand	Generic	
Beta Blocker	Brand	Generic	
Statin	Brand	Brand	
RAAS Blocker	Brand	Generic	
Price	285	154	
Adapted Price			

CHOICE SET 7

Attribute	Alternative 1	Alternative 2	Status Quo
Aspirin	Generic	Brand	
Beta Blocker	Brand	Generic	
Statin	Generic	Generic	
RAAS Blocker	Brand	Generic	
Price	109	22	
Adapted Price			

CHOICE SET 8

Attribute	Alternative 1	Alternative 2	Status Quo
Aspirin	Generic	Brand	
Beta Blocker	Generic	Brand	
Statin	Brand	Generic	
RAAS Blocker	Brand	Generic	
Price	222	63	
Adapted Price			

CHOICE SET 9

Attribute	Alternative 1	Alternative 2	Status Quo
Aspirin	Generic	Generic	
Beta Blocker	Generic	Brand	
Statin	Generic	Brand	
RAAS Blocker	Brand	Generic	
Price	68	195	
Adapted Price			

CHOICE SET 10

Attribute	Alternative 1	Alternative 2	Status Quo
Aspirin	Generic	Brand	
Beta Blocker	Brand	Generic	
Statin	Brand	Generic	
RAAS Blocker	Generic	Generic	
Price	195	22	
Adapted Price			

BLOCK 11

CHOICE SET 1

Attribute	Alternative 1	Alternative 2	Status Quo
Aspirin	Generic	Brand	
Beta Blocker	Generic	Brand	
Statin	Brand	Generic	
RAAS Blocker	Brand	Generic	
Price	222	63	
Adapted Price			

CHOICE SET 2

Attribute	Alternative 1	Alternative 2	Status Quo
Aspirin	Generic	Brand	
Beta Blocker	Brand	Generic	
Statin	Generic	Brand	
RAAS Blocker	Brand	Generic	
Price	109	176	
Adapted Price			

CHOICE SET 3

Attribute	Alternative 1	Alternative 2	Status Quo
Aspirin	Generic	Brand	
Beta Blocker	Brand	Generic	
Statin	Brand	Generic	
RAAS Blocker	Brand	Generic	
Price	263	22	
Adapted Price			

CHOICE SET 4

Attribute	Alternative 1	Alternative 2	Status Quo
Aspirin	Brand	Generic	
Beta Blocker	Brand	Generic	
Statin	Brand	Generic	
RAAS Blocker	Brand	Brand	
Price	285	68	
Adapted Price			

CHOICE SET 5

Attribute	Alternative 1	Alternative 2	Status Quo
Aspirin	Generic	Brand	
Beta Blocker	Generic	Generic	
Statin	Brand	Generic	
RAAS Blocker	Brand	Generic	
Price	222	22	
Adapted Price			

CHOICE SET 6

Attribute	Alternative 1	Alternative 2	Status Quo
Aspirin	Generic	Generic	
Beta Blocker	Generic	Brand	
Statin	Generic	Brand	
RAAS Blocker	Brand	Generic	
Price	68	195	
Adapted Price			

CHOICE SET 7

Attribute	Alternative 1	Alternative 2	Status Quo
Aspirin	Brand	Brand	
Beta Blocker	Brand	Generic	
Statin	Brand	Generic	
RAAS Blocker	Brand	Generic	
Price	285	22	
Adapted Price			

CHOICE SET 8

Attribute	Alternative 1	Alternative 2	Status Quo
Aspirin	Brand	Generic	
Beta Blocker	Generic	Brand	
Statin	Brand	Generic	
RAAS Blocker	Generic	Generic	
Price	176	41	
Adapted Price			

CHOICE SET 9

Attribute	Alternative 1	Alternative 2	Status Quo
Aspirin	Brand	Generic	
Beta Blocker	Brand	Brand	
Statin	Brand	Generic	
RAAS Blocker	Brand	Generic	
Price	285	41	
Adapted Price			

CHOICE SET 10

Attribute	Alternative 1	Alternative 2	Status Quo
Aspirin	Brand	Generic	
Beta Blocker	Generic	Brand	
Statin	Brand	Generic	
RAAS Blocker	Brand	Generic	
Price	244	41	
Adapted Price			

BLOCK 12

CHOICE SET 1

Attribute	Alternative 1	Alternative 2	Status Quo
Aspirin	Brand	Generic	
Beta Blocker	Brand	Generic	
Statin	Generic	Brand	
RAAS Blocker	Brand	Generic	
Price	131	154	
Adapted Price			

CHOICE SET 2

Attribute	Alternative 1	Alternative 2	Status Quo
Aspirin	Brand	Generic	
Beta Blocker	Brand	Generic	
Statin	Generic	Generic	
RAAS Blocker	Brand	Generic	
Price	131	0	
Adapted Price			

CHOICE SET 3

Attribute	Alternative 1	Alternative 2	Status Quo
Aspirin	Generic	Generic	
Beta Blocker	Brand	Generic	
Statin	Brand	Generic	
RAAS Blocker	Brand	Generic	
Price	263	0	
Adapted Price			

CHOICE SET 4

Attribute	Alternative 1	Alternative 2	Status Quo
Aspirin	Brand	Generic	
Beta Blocker	Generic	Brand	
Statin	Generic	Brand	
RAAS Blocker	Brand	Generic	
Price	90	195	
Adapted Price			

CHOICE SET 5

Attribute	Alternative 1	Alternative 2	Status Quo
Aspirin	Generic	Brand	
Beta Blocker	Generic	Brand	
Statin	Brand	Generic	
RAAS Blocker	Brand	Brand	
Price	222	131	
Adapted Price			

CHOICE SET 6

Attribute	Alternative 1	Alternative 2	Status Quo
Aspirin	Generic	Brand	
Beta Blocker	Brand	Generic	
Statin	Generic	Generic	
RAAS Blocker	Brand	Generic	
Price	109	22	
Adapted Price			

CHOICE SET 7

Attribute	Alternative 1	Alternative 2	Status Quo
Aspirin	Generic	Brand	
Beta Blocker	Brand	Generic	
Statin	Brand	Generic	
RAAS Blocker	Generic	Generic	
Price	195	22	
Adapted Price			

CHOICE SET 8

Attribute	Alternative 1	Alternative 2	Status Quo
Aspirin	Brand	Generic	
Beta Blocker	Brand	Generic	
Statin	Brand	Brand	
RAAS Blocker	Brand	Generic	
Price	285	154	
Adapted Price			

CHOICE SET 9

Attribute	Alternative 1	Alternative 2	Status Quo
Aspirin	Generic	Brand	
Beta Blocker	Brand	Brand	
Statin	Brand	Brand	
RAAS Blocker	Brand	Generic	
Price	263	217	
Adapted Price			

CHOICE SET 10

Attribute	Alternative 1	Alternative 2	Status Quo
Aspirin	Brand	Brand	
Beta Blocker	Generic	Generic	
Statin	Generic	Brand	
RAAS Blocker	Brand	Generic	
Price	90	176	
Adapted Price			

BLOCK 13

CHOICE SET 1

Attribute	Alternative 1	Alternative 2	Status Quo
Aspirin	Generic	Brand	
Beta Blocker	Generic	Brand	
Statin	Brand	Generic	
RAAS Blocker	Brand	Brand	
Price	222	131	
Adapted Price			

CHOICE SET 2

Attribute	Alternative 1	Alternative 2	Status Quo
Aspirin	Brand	Generic	
Beta Blocker	Generic	Brand	
Statin	Brand	Generic	
RAAS Blocker	Generic	Generic	
Price	176	41	
Adapted Price			

CHOICE SET 3

Attribute	Alternative 1	Alternative 2	Status Quo
Aspirin	Brand	Generic	
Beta Blocker	Generic	Brand	
Statin	Brand	Generic	
RAAS Blocker	Brand	Generic	
Price	244	41	
Adapted Price			

CHOICE SET 4

Attribute	Alternative 1	Alternative 2	Status Quo
Aspirin	Brand	Brand	
Beta Blocker	Generic	Generic	
Statin	Generic	Brand	
RAAS Blocker	Brand	Generic	
Price	90	176	
Adapted Price			

CHOICE SET 5

Attribute	Alternative 1	Alternative 2	Status Quo
Aspirin	Generic	Generic	
Beta Blocker	Brand	Generic	
Statin	Brand	Generic	
RAAS Blocker	Brand	Generic	
Price	263	0	
Adapted Price			

CHOICE SET 6

Attribute	Alternative 1	Alternative 2	Status Quo
Aspirin	Brand	Generic	
Beta Blocker	Brand	Brand	
Statin	Brand	Generic	
RAAS Blocker	Brand	Generic	
Price	285	41	
Adapted Price			

CHOICE SET 7

Attribute	Alternative 1	Alternative 2	Status Quo
Aspirin	Generic	Brand	
Beta Blocker	Brand	Generic	
Statin	Brand	Generic	
RAAS Blocker	Brand	Generic	
Price	263	22	
Adapted Price			

CHOICE SET 8

Attribute	Alternative 1	Alternative 2	Status Quo
Aspirin	Generic	Brand	
Beta Blocker	Brand	Brand	
Statin	Brand	Brand	
RAAS Blocker	Brand	Generic	
Price	263	217	
Adapted Price			

CHOICE SET 9

Attribute	Alternative 1	Alternative 2	Status Quo
Aspirin	Brand	Generic	
Beta Blocker	Brand	Generic	
Statin	Generic	Brand	
RAAS Blocker	Brand	Generic	
Price	131	154	
Adapted Price			

CHOICE SET 10

Attribute	Alternative 1	Alternative 2	Status Quo
Aspirin	Brand	Generic	
Beta Blocker	Generic	Brand	
Statin	Generic	Brand	
RAAS Blocker	Brand	Generic	
Price	90	195	
Adapted Price			

BLOCK 14

CHOICE SET 1

Attribute	Alternative 1	Alternative 2	Status Quo
Aspirin	Brand	Generic	
Beta Blocker	Brand	Generic	
Statin	Brand	Generic	
RAAS Blocker	Brand	Brand	
Price	285	68	
Adapted Price			

CHOICE SET 2

Attribute	Alternative 1	Alternative 2	Status Quo
Aspirin	Brand	Generic	
Beta Blocker	Brand	Generic	
Statin	Brand	Brand	
RAAS Blocker	Brand	Generic	
Price	285	154	
Adapted Price			

CHOICE SET 3

Attribute	Alternative 1	Alternative 2	Status Quo
Aspirin	Generic	Brand	
Beta Blocker	Brand	Generic	
Statin	Brand	Generic	
RAAS Blocker	Generic	Generic	
Price	195	22	
Adapted Price			

CHOICE SET 4

Attribute	Alternative 1	Alternative 2	Status Quo
Aspirin	Generic	Brand	
Beta Blocker	Generic	Generic	
Statin	Brand	Generic	
RAAS Blocker	Brand	Generic	
Price	222	22	
Adapted Price			

CHOICE SET 5

Attribute	Alternative 1	Alternative 2	Status Quo
Aspirin	Brand	Generic	
Beta Blocker	Brand	Generic	
Statin	Generic	Generic	
RAAS Blocker	Brand	Generic	
Price	131	0	
Adapted Price			

CHOICE SET 6

Attribute	Alternative 1	Alternative 2	Status Quo
Aspirin	Generic	Brand	
Beta Blocker	Brand	Generic	
Statin	Generic	Generic	
RAAS Blocker	Brand	Generic	
Price	109	22	
Adapted Price			

CHOICE SET 7

Attribute	Alternative 1	Alternative 2	Status Quo
Aspirin	Brand	Brand	
Beta Blocker	Brand	Generic	
Statin	Brand	Generic	
RAAS Blocker	Brand	Generic	
Price	285	22	
Adapted Price			

CHOICE SET 8

Attribute	Alternative 1	Alternative 2	Status Quo
Aspirin	Generic	Brand	
Beta Blocker	Brand	Generic	
Statin	Generic	Brand	
RAAS Blocker	Brand	Generic	
Price	109	176	
Adapted Price			

CHOICE SET 9

Attribute	Alternative 1	Alternative 2	Status Quo
Aspirin	Generic	Generic	
Beta Blocker	Generic	Brand	
Statin	Generic	Brand	
RAAS Blocker	Brand	Generic	
Price	68	195	
Adapted Price			

CHOICE SET 10

Attribute	Alternative 1	Alternative 2	Status Quo
Aspirin	Generic	Brand	
Beta Blocker	Generic	Brand	
Statin	Brand	Generic	
RAAS Blocker	Brand	Generic	
Price	222	63	
Adapted Price			

BLOCK 15

CHOICE SET 1

Attribute	Alternative 1	Alternative 2	Status Quo
Aspirin	Generic	Brand	
Beta Blocker	Brand	Generic	
Statin	Brand	Generic	
RAAS Blocker	Brand	Generic	
Price	263	22	
Adapted Price			

CHOICE SET 2

Attribute	Alternative 1	Alternative 2	Status Quo
Aspirin	Generic	Brand	
Beta Blocker	Brand	Generic	
Statin	Brand	Generic	
RAAS Blocker	Generic	Generic	
Price	195	22	
Adapted Price			

CHOICE SET 3

Attribute	Alternative 1	Alternative 2	Status Quo
Aspirin	Generic	Generic	
Beta Blocker	Generic	Brand	
Statin	Generic	Brand	
RAAS Blocker	Brand	Generic	
Price	68	195	
Adapted Price			

CHOICE SET 4

Attribute	Alternative 1	Alternative 2	Status Quo
Aspirin	Generic	Brand	
Beta Blocker	Generic	Brand	
Statin	Brand	Generic	
RAAS Blocker	Brand	Generic	
Price	222	63	
Adapted Price			

CHOICE SET 5

Attribute	Alternative 1	Alternative 2	Status Quo
Aspirin	Brand	Generic	
Beta Blocker	Brand	Brand	
Statin	Brand	Generic	
RAAS Blocker	Brand	Generic	
Price	285	41	
Adapted Price			

CHOICE SET 6

Attribute	Alternative 1	Alternative 2	Status Quo
Aspirin	Brand	Generic	
Beta Blocker	Brand	Generic	
Statin	Brand	Brand	
RAAS Blocker	Brand	Generic	
Price	285	154	
Adapted Price			

CHOICE SET 7

Attribute	Alternative 1	Alternative 2	Status Quo
Aspirin	Brand	Brand	
Beta Blocker	Generic	Generic	
Statin	Generic	Brand	
RAAS Blocker	Brand	Generic	
Price	90	176	
Adapted Price			

CHOICE SET 8

Attribute	Alternative 1	Alternative 2	Status Quo
Aspirin	Brand	Generic	
Beta Blocker	Generic	Brand	
Statin	Brand	Generic	
RAAS Blocker	Brand	Generic	
Price	244	41	
Adapted Price			

CHOICE SET 9

Attribute	Alternative 1	Alternative 2	Status Quo
Aspirin	Generic	Brand	
Beta Blocker	Generic	Brand	
Statin	Brand	Generic	
RAAS Blocker	Brand	Brand	
Price	222	131	
Adapted Price			

CHOICE SET 10

Attribute	Alternative 1	Alternative 2	Status Quo
Aspirin	Brand	Generic	
Beta Blocker	Brand	Generic	
Statin	Generic	Generic	
RAAS Blocker	Brand	Generic	
Price	131	0	
Adapted Price			

BLOCK 16

CHOICE SET 1

Attribute	Alternative 1	Alternative 2	Status Quo
Aspirin	Brand	Generic	
Beta Blocker	Generic	Brand	
Statin	Brand	Generic	
RAAS Blocker	Generic	Generic	
Price	176	41	
Adapted Price			

CHOICE SET 2

Attribute	Alternative 1	Alternative 2	Status Quo
Aspirin	Generic	Brand	
Beta Blocker	Brand	Generic	
Statin	Generic	Brand	
RAAS Blocker	Brand	Generic	
Price	109	176	
Adapted Price			

CHOICE SET 3

Attribute	Alternative 1	Alternative 2	Status Quo
Aspirin	Generic	Brand	
Beta Blocker	Brand	Generic	
Statin	Generic	Generic	
RAAS Blocker	Brand	Generic	
Price	109	22	
Adapted Price			

CHOICE SET 4

Attribute	Alternative 1	Alternative 2	Status Quo
Aspirin	Generic	Brand	
Beta Blocker	Brand	Brand	
Statin	Brand	Brand	
RAAS Blocker	Brand	Generic	
Price	263	217	
Adapted Price			

CHOICE SET 5

Attribute	Alternative 1	Alternative 2	Status Quo
Aspirin	Brand	Brand	
Beta Blocker	Brand	Generic	
Statin	Brand	Generic	
RAAS Blocker	Brand	Generic	
Price	285	22	
Adapted Price			

CHOICE SET 6

Attribute	Alternative 1	Alternative 2	Status Quo
Aspirin	Brand	Generic	
Beta Blocker	Brand	Generic	
Statin	Generic	Brand	
RAAS Blocker	Brand	Generic	
Price	131	154	
Adapted Price			

CHOICE SET 7

Attribute	Alternative 1	Alternative 2	Status Quo
Aspirin	Brand	Generic	
Beta Blocker	Brand	Generic	
Statin	Brand	Generic	
RAAS Blocker	Brand	Brand	
Price	285	68	
Adapted Price			

CHOICE SET 8

Attribute	Alternative 1	Alternative 2	Status Quo
Aspirin	Generic	Generic	
Beta Blocker	Brand	Generic	
Statin	Brand	Generic	
RAAS Blocker	Brand	Generic	
Price	263	0	
Adapted Price			

CHOICE SET 9

Attribute	Alternative 1	Alternative 2	Status Quo
Aspirin	Brand	Generic	
Beta Blocker	Generic	Brand	
Statin	Generic	Brand	
RAAS Blocker	Brand	Generic	
Price	90	195	
Adapted Price			

CHOICE SET 10

Attribute	Alternative 1	Alternative 2	Status Quo
Aspirin	Generic	Brand	
Beta Blocker	Generic	Generic	
Statin	Brand	Generic	
RAAS Blocker	Brand	Generic	
Price	222	22	
Adapted Price			
