# Supplementary material for: Uncovering patients’ preferences for brand among essential classes of coronary heart disease medications using a discrete choice experiment
Source: Sci Rep. 2024 Nov 4;14:26643. doi: 10.1038/s41598-024-77007-3 (PMC11535387; doi:10.1038/s41598-024-77007-3)
Supplement: Supplementary file 2 — Supplementary Information 2. [file 41598_2024_77007_MOESM2_ESM.pdf]

Direct Survey Instrument

1. To what extent are you satisfied with your dispensed generic (free-of-charge) medicines? ما مدي رضائك عن الأدوية الجنيسة المجانية التي يتم صرفها لك؟

Mark only one oval per row.

	I accept it and I am satisfied أقبله و راض عنه	I would like to change it but I cannot afford أود تغييره لكن لا يمكنني تحمل التكلفة	I changed it to a brand لقد غيرته الي منتج ذي علامة تجارية	Not Prescribed غير موصوف
**Aspirin**	⬭	⬭	⬭	⬭
**Beta Blocker**	⬭	⬭	⬭	⬭
**Statin**	⬭	⬭	⬭	⬭
**RAAS Blocker**	⬭	⬭	⬭	⬭

2. What drove you to buy or wish to buy the brand as per your answers in the previous question? ما الذي دفعك إلي شراء المنتجات ذات العلامات التجارية أو الرغبة في شرائها وفقا لاجاباتك في السؤال السابق

Mark only one oval per row.

	I answered I accept it and I am satisfied جاوبت بأني "أقبله و راض عنه"	I tried the generic but I found it not effective لقد جربت المنتج الجنيس المجاني لكنني وجدته غير فعال	Advice of my health insurance doctor بناء علي نصيحة من طبيب التأمين الصحي الخاص بي	Advice of my private doctor (outside of health insurance) بناء علي نصيحة من طبيب خاص خارج التأمين الصحي	Advice of a friend or relative بناء علي نصيحة من صديق أو قريب	Other سبب آخر	Not Prescribed غير موصوف
**Aspirin**	◯	◯	◯	◯	◯	◯	◯
**Beta Blocker**	◯	◯	◯	◯	◯	◯	◯
**Statin**	◯	◯	◯	◯	◯	◯	◯
**RAAS Blocker**	◯	◯	◯	◯	◯	◯	◯

This content is neither created nor endorsed by Google.

Google Forms
