# Supplementary material for: Uncovering patients’ preferences for brand among essential classes of coronary heart disease medications using a discrete choice experiment
Source: Sci Rep. 2024 Nov 4;14:26643. doi: 10.1038/s41598-024-77007-3 (PMC11535387; doi:10.1038/s41598-024-77007-3)
Supplement: Supplementary file 3 — Supplementary Information 3. [file 41598_2024_77007_MOESM3_ESM.docx]

**Appendix I (Analysis by Age)**

| Brands' preference weights and the adjusted odds ratios for preferring each class’s brand over its generic counterpart while holding the other classes constant (**Analysis by age)** | | | | | | | | |
| --- | --- | --- | --- | --- | --- | --- | --- | --- |
| **Attribute**  **(brands)** | ***β*** | **95 % CI** | | **AOR** | **95 % CI** | | | **P** |
|  |  | **lower** | **upper** |  | **lower** | **upper** | |  |
|  | **Below 68 years old (n = 70)** | | | | | |  |  |
|  |  |  |  |  |  |  |  |  |
| **Aspirin** | -0.32 | -0.45 | -0.18 | **0.73** | **0.64** | **0.83** | | **< .001*** |
| **Beta-blocker** | 0.72 | 0.58 | 0.85 | **2.05** | **1.79** | **2.34** | | **< .001*** |
| **Statin** | 0.49 | 0.32 | 0.66 | **1.63** | **1.38** | **1.94** | | **< .001*** |
| **RAAS blocker** | 1.05 | 0.90 | 1.20 | **2.86** | **2.47** | **3.32** | | **< .001*** |
| **Price** | -0.01 | -0.01 | -0.01 | **0.99** | **0.99** | **0.99** | | **< .001*** |
|  | **68 years old and above (n = 79)** | | | | | |  |  |
|  |  |  |  |  |  |  |  |  |
| **Aspirin** | -0.35 | -0.49 | -0.22 | **0.70** | **0.61** | **0.80** | | **< .001*** |
| **Beta-blocker** | 0.76 | 0.63 | 0.89 | **2.14** | **1.88** | **2.45** | | **< .001*** |
| **Statin** | 0.30 | 0.14 | 0.46 | **1.35** | **1.15** | **1.58** | | **< .001*** |
| **RAAS blocker** | 1.27 | 1.12 | 1.41 | **3.55** | **3.06** | **4.11** | | **< .001*** |
| **Price** | -0.01 | -0.01 | -0.01 | **0.99** | **0.99** | **0.99** | | **< .001*** |

**AOR** indicates adjusted odds ratio for preferring a class’s brand over its generic counterpart because it provides a higher utility from the perspective of the 149 dissatisfied patients (with at least one of the generic). ***β*** indicates preference weight. **95% CI** indicates 95% confidence interval.

**Appendix II (Analysis by Education)**

| Brands' preference weights and the adjusted odds ratios for preferring each class’s brand over its generic counterpart while holding the other classes constant (**Analysis by education)** | | | | | | | | |
| --- | --- | --- | --- | --- | --- | --- | --- | --- |
| **Attribute**  **(brands)** | ***β*** | **95 % CI** | | **AOR** | **95 % CI** | | | **P** |
|  |  | **lower** | **upper** |  | **lower** | **upper** | |  |
|  | **At least intermediate education (n = 88)** | | | | | |  |  |
|  |  |  |  |  |  |  |  |  |
| **Aspirin** | -0.31 | -0.43 | -0.20 | **0.73** | **0.65** | **0.82** | | **< .001*** |
| **Beta-blocker** | 0.81 | 0.69 | 0.93 | **2.25** | **2.00** | **2.54** | | **< .001*** |
| **Statin** | 0.18 | 0.03 | 0.33 | **1.19** | **1.03** | **1.38** | | **.018*** |
| **RAAS blocker** | 0.99 | 0.86 | 1.12 | **2.70** | **2.37** | **3.08** | | **< .001*** |
| **Price** | 0.00 | -0.01 | 0.00 | **1.00** | **0.99** | **1.00** | | **< .001*** |
|  | **Below intermediate education (n = 61)** | | | | | |  |  |
|  |  |  |  |  |  |  |  |  |
| **Aspirin** | -0.25 | -0.42 | -0.08 | **0.78** | **0.66** | **0.92** | | **.004*** |
| **Beta-blocker** | 0.74 | 0.57 | 0.91 | **2.09** | **1.76** | **2.47** | | **< .001*** |
| **Statin** | 0.78 | 0.58 | 0.98 | **2.17** | **1.78** | **2.66** | | **< .001*** |
| **RAAS blocker** | 1.57 | 1.38 | 1.76 | **4.82** | **3.98** | **5.84** | | **< .001*** |
| **Price** | -0.02 | -0.02 | -0.02 | **0.98** | **0.98** | **0.98** | | **< .001*** |

**AOR** indicates adjusted odds ratio for preferring a class’s brand over its generic counterpart because it provides a higher utility from the perspective of the 149 dissatisfied patients (with at least one of the generic). ***β*** indicates preference weight. **95% CI** indicates 95% confidence interval.

**Appendix III (Analysis by Income)**

| Brands' preference weights and the adjusted odds ratios for preferring each class’s brand over its generic counterpart while holding the other classes constant (**Analysis by income)** | | | | | | | | |
| --- | --- | --- | --- | --- | --- | --- | --- | --- |
| **Attribute**  **(brands)** | ***β*** | **95 % CI** | | **AOR** | **95 % CI** | | | **P** |
|  |  | **lower** | **upper** |  | **lower** | **upper** | |  |
|  | **In debt (n = 62)** | | | | | |  |  |
|  |  |  |  |  |  |  |  |  |
| **Aspirin** | -0.36 | -0.53 | -0.20 | **0.69** | **0.59** | **0.82** | | **< .001*** |
| **Beta-blocker** | 0.84 | 0.68 | 1.01 | **2.33** | **1.97** | **2.75** | | **< .001*** |
| **Statin** | 1.02 | 0.82 | 1.22 | **2.78** | **2.28** | **3.39** | | **< .001*** |
| **RAAS blocker** | 1.23 | 1.05 | 1.41 | **3.42** | **2.86** | **4.10** | | **< .001*** |
| **Price** | -0.02 | -0.02 | -0.02 | **0.98** | **0.98** | **0.98** | | **< .001*** |
|  | **Not in debt (n = 87)** | | | | | |  |  |
|  |  |  |  |  |  |  |  |  |
| **Aspirin** | -0.31 | -0.43 | -0.19 | **0.73** | **0.65** | **0.83** | | **< .001*** |
| **Beta-blocker** | 0.74 | 0.62 | 0.86 | **2.10** | **1.86** | **2.37** | | **< .001*** |
| **Statin** | 0.07 | -0.08 | 0.22 | **1.07** | **0.92** | **1.24** | | .368 |
| **RAAS blocker** | 1.19 | 1.06 | 1.33 | **3.30** | **2.88** | **3.78** | | **< .001*** |
| **Price** | 0.00 | -0.01 | 0.00 | **1.00** | **0.99** | **1.00** | | **< .001*** |

**AOR** indicates adjusted odds ratio for preferring a class’s brand over its generic counterpart because it provides a higher utility from the perspective of the 149 dissatisfied patients (with at least one of the generic). ***β*** indicates preference weight. **95% CI** indicates 95% confidence interval.

**Appendix IV: Relative importance by age, education, and income**


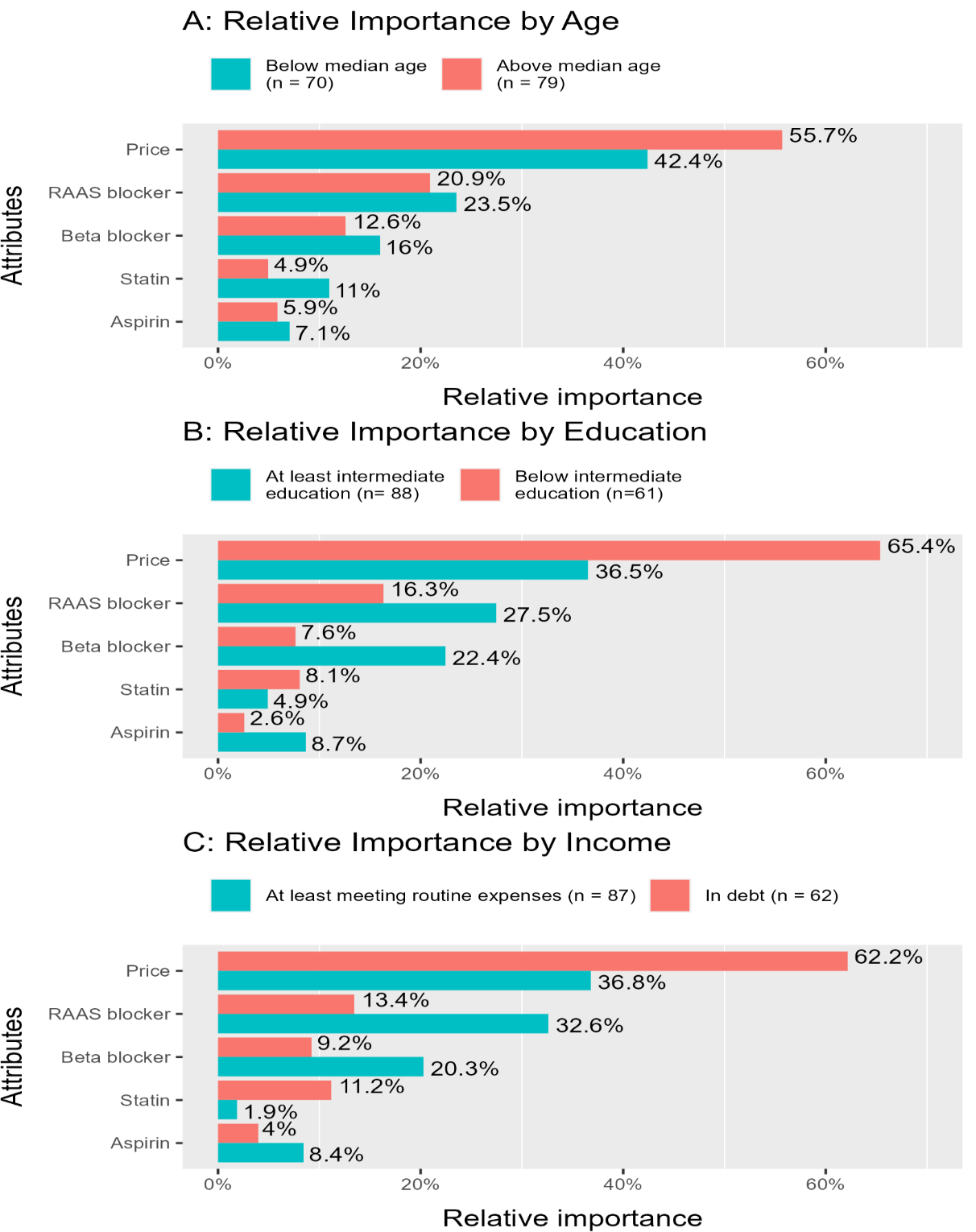


**Appendix V (Levels of Satisfaction with the generics)**

| **Levels of patients' acceptance of using the dispensed generic cardiovascular drugs (n (%))** | | | |
| --- | --- | --- | --- |
| CHD drug class | Satisfied with the generic | Brand inclined | |
|  |  | Intention to change | Changed to brand |
| **Aspirin** | 50 (34%) | 81 (54%) | 18 (12%) |
| **Beta-Blocker** | 30 (20%) | 69 (46%) | 50 (34%) |
| **Statin** | 41 (28%) | 95 (63%) | 13 (9%) |
| **RAAS Blocker** | 22 (15%) | 75 (50%) | 52 (35%) |

**Appendix VI (Drivers for Brand Inclinations)**

| **Drivers for inclination in brand-inclined patients (n (%))** | | | | |
| --- | --- | --- | --- | --- |
|  | **Aspirin**  **(n = 99)** | **Beta-Blocker (n = 119)** | **Statin**  **(n = 108)** | **RAAS Blocker (n =127)** |
| **Internal Drivers** | **91 (91.9%)** | **95 (79.8%)** | **98 (90.7%)** | **96 (75.6%)** |
| I tried the generic, but I found it not effective | 54 (54.5%) | 65 (54.6%) | 64 (59.3%) | 67 (52.8%) |
| I believe any brand is better | 35 (35.4%) | 30 (25.2%) | 34 (31.5%) | 29 (22.8%) |
| The generic causes me side effects | 2 (2.0%) | 0 (0%) | 0 (0%) | 0 (0%) |
| **External Drivers** | **8 (8.1%)** | **24 (20.2%)** | **10 (9.3%)** | **31 (24.4%)** |
| The advice of my health insurance doctor | 1 (1.0%) | 8 (6.7%) | 1 (0.9%) | 13 (10.2%) |
| The advice of my private doctor (outside of health insurance) | 6 (6.1%) | 15 (12.6%) | 8 (7.4%) | 17 (13.4%) |
| The advice of a friend or relative | 1 (1.0%) | 1 (0.8%) | 1 (0.9%) | 1 (0.8%) |

**Appendix VII (Detailed sociodemographic)**

**Appendix VIII (Analysis by sex)**

| Brands' preference weights and the adjusted odds ratios for preferring each class’s brand over its generic counterpart while holding the other classes constant (**Analysis by sex)** | | | | | | | | |
| --- | --- | --- | --- | --- | --- | --- | --- | --- |
| **Attribute**  **(brands)** | ***β*** | **95 % CI** | | **AOR** | **95 % CI** | | | **P** |
|  |  | **lower** | **upper** |  | **lower** | **upper** | |  |
|  | **Male (n = 114)** | | | | | |  |  |
|  |  |  |  |  |  |  |  |  |
| **Aspirin** | -0.29 | -0.39 | -0.18 | **0.75** | **0.68** | **0.84** | | **< .001*** |
| **Beta-blocker** | 0.78 | 0.68 | 0.89 | **2.19** | **1.97** | **2.44** | | **< .001*** |
| **Statin** | 0.44 | 0.30 | 0.57 | **1.55** | **1.36** | **1.76** | | **< .001*** |
| **RAAS blocker** | 1.09 | 0.97 | 1.21 | **2.99** | **2.65** | **3.36** | | **< .001*** |
| **Price** | -0.01 | -0.01 | -0.01 | **0.99** | **0.99** | **0.99** | | **< .001*** |
|  | **Female (n = 35)** | | | | | |  |  |
|  |  |  |  |  |  |  |  |  |
| **Aspirin** | -0.51 | -0.70 | -0.31 | **0.60** | **0.49** | **0.74** | | **< .001*** |
| **Beta-blocker** | 0.57 | 0.38 | 0.76 | **1.77** | **1.46** | **2.14** | | **< .001*** |
| **Statin** | 0.31 | 0.07 | 0.55 | **1.36** | **1.07** | **1.74** | | **.013*** |
| **RAAS blocker** | 1.33 | 1.12 | 1.55 | **3.79** | **3.06** | **4.71** | | **< .001*** |
| **Price** | -0.01 | -0.01 | -0.01 | **0.99** | **0.99** | **0.99** | | **< .001*** |

**AOR** indicates adjusted odds ratio for preferring a class’s brand over its generic counterpart because it provides a higher utility from the perspective of the 149 dissatisfied patients (with at least one of the generic). ***β*** indicates preference weight. **95% CI** indicates 95% confidence interval.
