# Supplementary material for: Uncovering patients’ preferences for brand among essential classes of coronary heart disease medications using a discrete choice experiment
Source: Sci Rep. 2024 Nov 4;14:26643. doi: 10.1038/s41598-024-77007-3 (PMC11535387; doi:10.1038/s41598-024-77007-3)
Supplement: Supplementary file 4 — Supplementary Information 4. [file 41598_2024_77007_MOESM4_ESM.pdf]

Sociodemographic Characteristics

الخصائص الاجتماعية الديموغرافية

1. Age (years) العمر

____

2. Sex الجنس

Mark only one oval.

- ( ) Male ذكر
- ( ) Female انثي

3. Highest level of education أعلي مستوي تعليمي

Mark only one oval.

- ( ) Illiterate أمي
- ( ) Read & write أقرأ و أكتب
- ( ) Primary ابتدائي
- ( ) Preparatory إعدادي
- ( ) Secondary (general & technical of 3 or 5 years) ثانوي عام أو فني (من 3-5 سنوات)
- ( ) Intermediate institute (2 years) معهد متوسط (سنتين)
- ( ) University graduate شهادة جامعية
- ( ) Postgraduate degree دراسات عليا

4. Occupation المهنة

Mark only one oval.

- Non-working / house wife لا يعمل / ربة منزل
- Unskilled manual worker عامل بأعمال يدوية غير فنية
- Skilled manual worker / farmer عامل فني / مزارع
- Trades / business التجارة
- Semi-professional / clerk أعمال كتابية / شبه حرفي
- Professional عمل حرفي

5. To what extent is your monthly income (from all sources) proportional to your expenses? الي أي مدي يتناسب دخلك الشهري (من كافة المصادر) مع نفقاتك؟

Mark only one oval.

- In debt مدين
- Just meet routine expenses فقط يلبي النفقات الروتينية
- Meet routine expenses and emergencies يلبي النفقات الروتينية و الطارئة
- Able to save/invest money قادر علي الادخار و الاستثمار

This content is neither created nor endorsed by Google.

Google Forms
